# Mining the tissue-tissue gene co-expression network for tumor microenvironment study and biomarker prediction

**DOI:** 10.1186/1471-2164-14-S5-S4

**Published:** 2013-10-16

**Authors:** Yang Xiang, Jie Zhang, Kun Huang

**Affiliations:** 1 Department of Biomedical Informatics, The Ohio State University, Columbus OH, USA

## Abstract

**Background:**

Recent discovery in tumor development indicates that the tumor microenvironment (mostly stroma cells) plays an important role in cancer development. To understand how the tumor microenvironment (TME) interacts with the tumor, we explore the correlation of the gene expressions between tumor and stroma. The tumor and stroma gene expression data are modeled as a weighted bipartite network (tumor-stroma coexpression network) where the weight of an edge indicates the correlation between the expression profiles of the corresponding tumor gene and stroma gene. In order to efficiently mine this weighted bipartite network, we developed the Bipartite subnetwork Component Mining algorithm (BCM), and we show that the BCM algorithm can efficiently mine weighted bipartite networks for dense Bipartite sub-Networks (BiNets) with density guarantees.

**Results:**

We applied BCM to the tumor-stroma coexpression network and find 372 BiNets that demonstrate statistical significance in survival tests. A good number of these BiNets demonstrate strong prognosis powers on at least one breast cancer patient cohort, which suggests that these BiNets are potential biomarkers for breast cancer prognosis. Further study on these 372 BiNets by the network merging approach reveals that they form 10 macro bipartite networks which show orchestrated key biological processes in both tumor and stroma. In addition, by further examining the BiNets that are significant in ER-negative breast cancer patient prognosis, we discovered a ubiquitin C (*UBC*) gene network that demonstrates strong prognosis power in nearly all types of breast cancer subtypes we used in this study.

**Conclusions:**

The results support our hypothesis that the *UBC *gene network plays an important role in breast cancer prognosis and therapy and it is a potential prognostic biomarker for multiple breast cancer subtypes.

## Introduction

The initiation, development and metastasis of tumor are complicated biological processes. The tumor microenvironment (TME), which surrounds the tumor immediately with secreted proteins, small signaling molecules, blood vessels, and normal cells, plays an essential role in each step. Tumor and its microenvironment consist of diverse cell types. For instance, for epithelial type of cancers, besides the epithelial cells, the TME includes fibroblast, endothelial cell, macrophage, and etc. All of them play critical roles in the formation and development of tumor [1]. In addition, recently it has been shown that genetic changes in the stroma (e.g., in fibroblast) can lead to the development of epithelial tumor [2]. Therefore, an important issue in cancer research is to understand how TME components interact with the tumor. It has been suggested that such interaction is mediated by extracellular molecules coded by the so-called *stromal genes *including signaling molecules such as cytokines/chemokines, structural molecules such as collagens (and the associated receptors such as DDR2) and extracellular proteinase such as metalloprotease (MMPs). It has been shown that some of these stromal genes may serve as important biomarkers to predicting drug responses for ER-negative breast cancer patients which are usually considered to have poor prognosis [3]. Recent research results provide further evidences that tumor-stroma interaction plays an important role in breast cancer tumor growth [4,5]. However, despite these progresses and intensive research efforts, many issues still remain unclear, including how such interactions lead to the intracellular changes in tumor and TME components.

Recently there has been a study using tissue-tissue gene co-expression network to characterize the interactions and the corresponding intracellular effects in obesity study [6]. Basically by identifying gene clusters that show high levels co-expression between different tissues, researchers discovered orchestrated biological processes between different tissues without the need to explicitly characterize the intercellular signaling mechanisms. In this paper, we adopted this approach to study the gene co-expression between tumor and its microenvironment in breast cancer. Specifically, we used a public gene expression microarray dataset consisting of 47 breast cancer biopsy samples, in which tumor and the matching surrounding stroma (TME) are isolated by laser capture microdissection (LCM) technology. The gene expression profiles in this dataset are generated for tumor and stroma separately for every sample [7]. By mining the tightly correlated gene expression profiles between the matching tumor and stroma, we identify dense networks of putative gene interactions between the two tissues (tumor and stroma). Our goals are to characterize orchestrated biological processes between the two tissues through the identified gene-gene communications/interactions, and at the same time to identify potential new biomarkers for breast cancer prognosis or treatment prediction.

From the bioinformatics point of view, our project falls into the category of gene co-expression network (GCN) analysis. GCN analysis has been shown to be very effective in discovering new gene functions [8], predicting disease biomarkers [9] and identifying disease genes [10]. However, most of GCN analysis methods focuse on a single type of sample. For tissue-tissue GCN, the problem was formulated as a bipartite graph mining problem in [6] in which a heuristic algorithm was used on a thresholded binary bipartite graph.

Mining dense components from bipartite graphs is a fundamental research problem in related fields. A simplest version of this problem is to find just one maximum clique in an unweighted bipartite graphs. Even for this simplest version, it was proved [11] to be an NP-hard problem. To tackle this problem, a few dense component mining algorithms, e.g. [12-15], having been proposed for unweighted bipartite graphs, for which many efficient pruning techniques are available. However, in biomedical research, many data are in the form of weighted bipartite graphs. Since the correlation coefficients between gene expression profiles can be used as weights of the edges in the graph, we expect a weighted bipartite graph mining approach would provide much more information on the gene-gene crosstalk between different tissue types. Given the successful cases of mining weighted network data [16,17], we want to extend our work to mine weighted *bipartite *networks in matching gene expression data from different tissue types. As a result, in this paper we propose a novel weighted Bipartite network Component Mining algorithm BCM which guarantees a lower bound on the densities of the identified components, i.e., Bipartite sub-Networks (BiNets). We tested and validated the prognosis power of identified BiNets on three separate breast cancer microarray studies. In addition, the results of BCM can be further summarized by our network merging approach which also guarantees a lower bound on the densities of summarized macro networks. We would like to point out that although clustering-based approaches such as [10,18,19] for gene co-expression network may be extended to handle weighted bipartite networks, our approach has clear advantages on exploring these networks for biomarker prediction. This is because BCM allows shared genes between BiNets and can find small dense BiNets that are suitable for biomarker prediction. At the same time, our approach is able to merge BiNets into Macro Bipartite Networks for understanding the general structure of the bipartite networks. Shared genes may still exist between Macro Bipartite Networks. In contrast, the clustering based approaches do not allow shared genes between two clusters and the clusters identified are often too large to find small gene networks with subtle functions.

## Results

### BiNets in tumor-stroma co-expression network

Using the BCM algorithm described in the Materials and Methods, we obtained 826 BiNets with a bounded density. Among them, 422 contain at least 10 distinct genes. These 422 BiNets were then subjected to survival analysis on five different breast cancer patient cohorts, i.e., the entire patients in the Netherlands Cancer Institute (NKI) dataset [20,21], the Lymph-Node-positive (LN-positive) patients in the NKI dataset, the Estrogen-Receptor-negative (ER-negative) patients in the NKI dataset, the entire patients in the GSE1456 (Stockholm) dataset [22], and the entire patients in the GSE2034 (Wang) dataset [23,24]. The results showed that 372 BiNets have significant prognostic power (p-values *<*0.05 from log-rank test) in at least one patient group. The percentage (372/422 ≈ 88.2%) demonstrates the effectiveness of mining tumor-stroma co-expression network using BCM. The number of BiNets with p-value less than 0.05 for each patient cohort is listed in Table 1, from which we can also observe that survival tests on three patient cohorts (NKI, NKI LN-Positive, GSE1456) yield a minimum p-value no more than 7.466*e *− 08.

**Table 1:** Log-rank test summary

	NKI data (295 patients)	NKI LN-positive (144 patients)	NKI ER-negative (69 patients)	GSE2034	GSE1456
Number of Bi-Nets with P-value*<*0.05	306	260	14	27	277

Minimum observed P-value	1.763*e *− 13	6.698*e *- 09	7.905*e *− 04	2.153e-03	7.466*e *− 08


Summary of the log-rank tests on patient groups or subtypes separated by genes in each BiNet.

**Figure 1:**
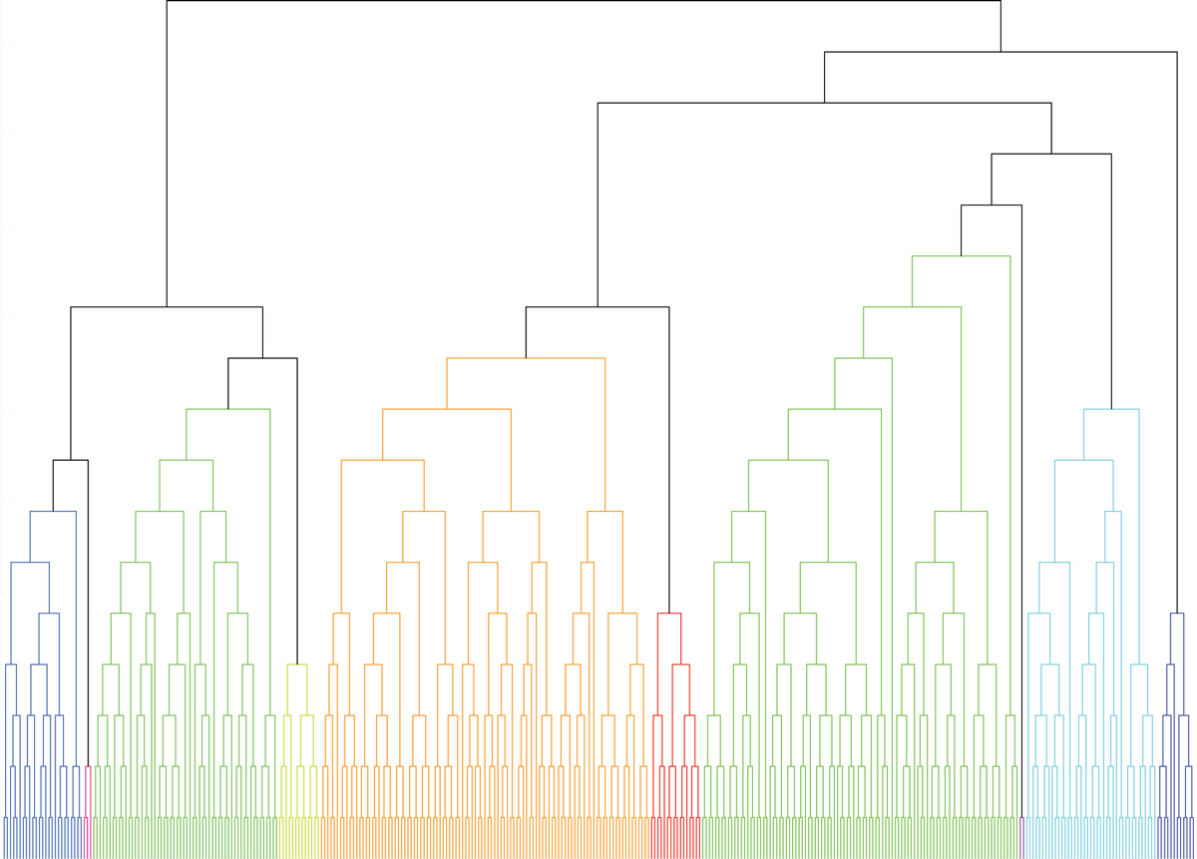
**Merging bipartite networks**. Merge the 372 BiNets into ten macro bipartite networks. The colors are for distinguishing different macro bipartite networks.

To obtain a macro view on these BiNets, we further merge the identified BiNets into larger clusters. Figure 1 shows the dendrogram of merging the 372 BiNets using [25]. At a density boundary of 0.3, the merging algorithm yields 10 macro bipartite networks and we further apply Gene Ontology enrichment analysis on these clusters by Toppgene (http://toppgene.cchmc.org/enrichment.jsp). Table 2 describes the most enriched GO term for each macro bipartite network.

**Table 2:** GO ontology enrichment analysis for ten macro bipartite networks

	BiNets	density	Tumor Genes	Top Enriched GO Terms (p-value)	Stroma Genes	Top Enrich GO Terms (p-value)
1	25	0.365644	118	BP: cardiovascular system development (2.309E-14); CC: extracellular matrix (1.098E-20)	154	BP: muscle organ development (5.371E-9); CC: extracellular matrix (8.853E-19)

2	3	0.534566	73	BP: adenylate cyclase-activating G-protein coupled receptor signaling pathway (1.547E-6)	4	BP: protein-chromophore linkage (2.271E-3)

3	58	0.332754	278	BP: response to iron ion (1.948E-5), epithelial cell development (4.760E-5), response to estrogen stimulus (2.065E-4)	224	BP: gland development (8.306E-6), development of primary male sexual characteristics (1.596E-5), male sex differentiation (2.602E-5); MF: enzyme binding (1.636E-4); CC cell projection (6.956E-8)

4	13	0.326076	113	BP: cell-cell signaling (1.934E-8); MF: receptor binding (8.154E-6)	89	BP: cell-cell signaling (2.233E-9); MF: receptor binding (5.040E-7)

5	103	0.320424	521	BP: mitotic cell cycle (3.640E-32), cell cycle phase (3.333E-30), cell cycle process (5.346E-27), cell cycle (4.043E-25); MF: RNA binding (3.610E-9)	629	BP: mitotic cell cycle (7.256E-41), cell cycle phase (9.357E-39), cell cycle process (1.771E-32), cell cycle (1.319E-28); MF: RNA binding (2.821E-11)

6	16	0.3014	117	BP: defense response to virus (2.621E-32), response to virus (2.914E-32); MF: double-stranded RNA binding (5.875E-12)	99	BP: defense response to virus (1.307E-34), response to virus (2.873E-33), innate immune response (2.983E-32); MF: double-stranded RNA binding (1.255E-12)

7	99	0.329521	525	BP: mitotic cell cycle (5.339E-29), cell cycle phase (3.764E-27), cell cycle process (1.512E-23); CC: mitochondrial part (9.680E-23)	489	BP: cell cycle phase (7.556E-34), mitotic cell cycle (4.042E-32), cell cycle process (1.732E-28); MF: RNA binding (1.179E-14); CC: mitochondrial part (5.769E-24)

8	2	0.464059	26	BP: response to progesterone stimulus (4.135E-4)	30	BP: immune response (1.287E-7)

9	41	0.348161	278	BP: respiratory electron transport chain (1.096E-28), electron transport chain (1.967E-24), cellular respiration (1.066E-23); CC: mitochondrial part (7.866E-24)	219	BP: respiratory electron transport chain (2.241E-27), cellular respiration (1.460E-25), electron transport chain (1.381E-23); MF: RNA binding (7.907E-16); CC: mitochondrial membrane part (1.505E-23), mitochondrial part (6.889E-23), mitochondrial inner membrane (2.245E-21), organelle inner membrane (2.951E-21)

10	12	0.417881	80	BP: immune response (7.503E-25)	110	BP: defense response (3.448E-22), immune response (4.224E-21)


For each macro bipartite network, we list the numbers of genes in the tumor side and the stroma side separately as well as significant GO terms with the p-values obtained from ToppGenes. P-values were before Bonferroni corrections. BP, MF, and CC stand for Biological Process, Molecular Function and Cellular Component, respectively.

### BiNets as potential biomarkers for breast cancer prognosis

**Figure 2:**
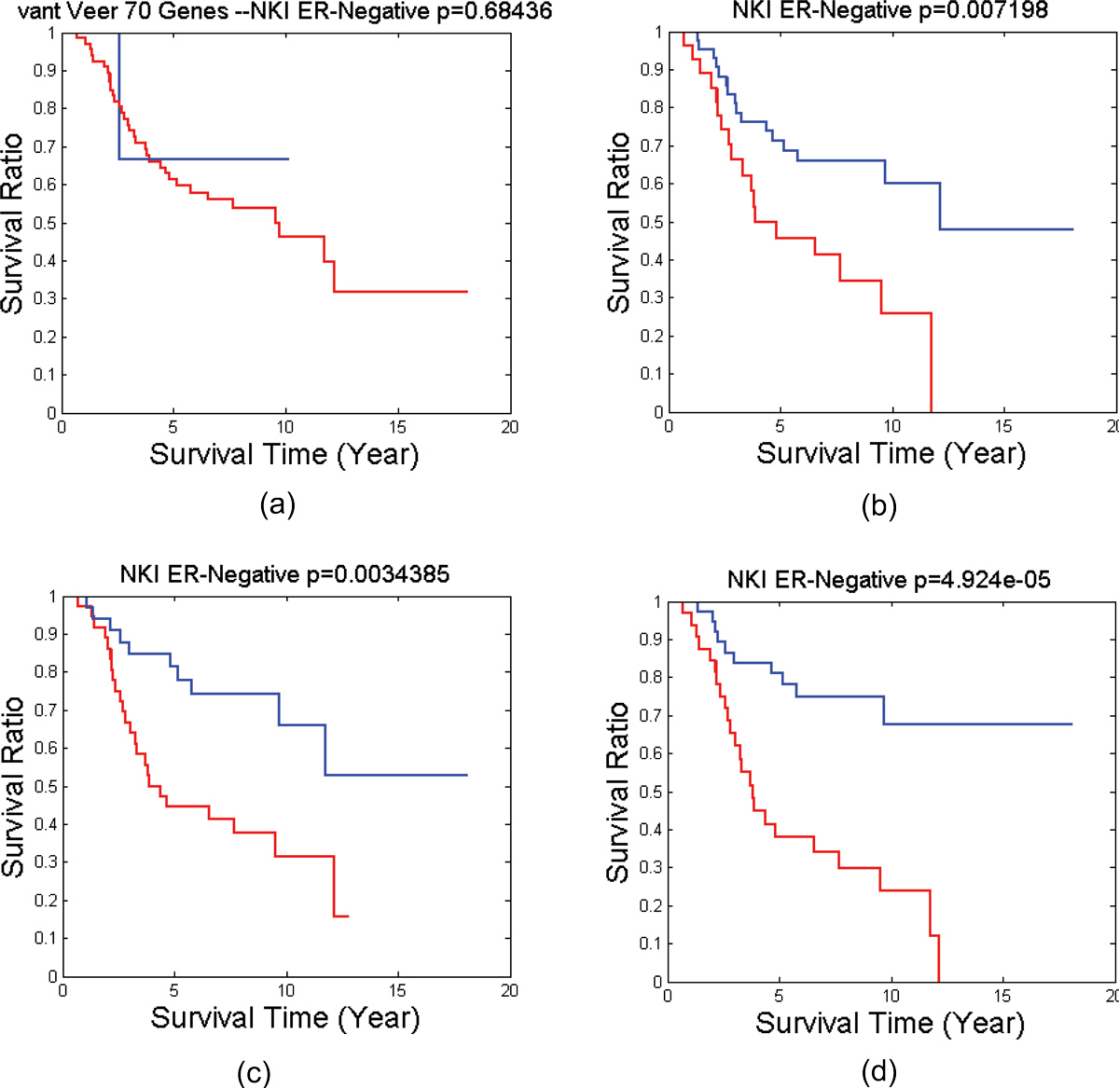
**Survival tests on NKI ER-Negative**. The survival test on NKI ER-Negative patients using (a) well-established 70-gene signature from [32], (b) Genes in BiNet 52 "C11orf51, DAP, EBP, HOMER2, LOC100129361, MAGT1, NDUFS6, NUDT21, PEX3, SDHA, SLC3A2", (c) Genes in BiNet 228 "C4BPB, CCR10, CKM, CPS1, CYP2F1, GPR6, GUCY1A2, HAUS6, HPD, HYAL1, PGAM2, PLA1A, PPP1R14D, PROC, REC8, SERPINA6, SFTPA2, STXBP5L, SYNPO2L, TGFB2, TPTE, VASH2", (d) the union of gene lists (b) and (c) plus gene *UBC*. Blue lines are the survival curves of good survival groups. Red lines are the survival curves of poor survival groups.

**Figure 3:**
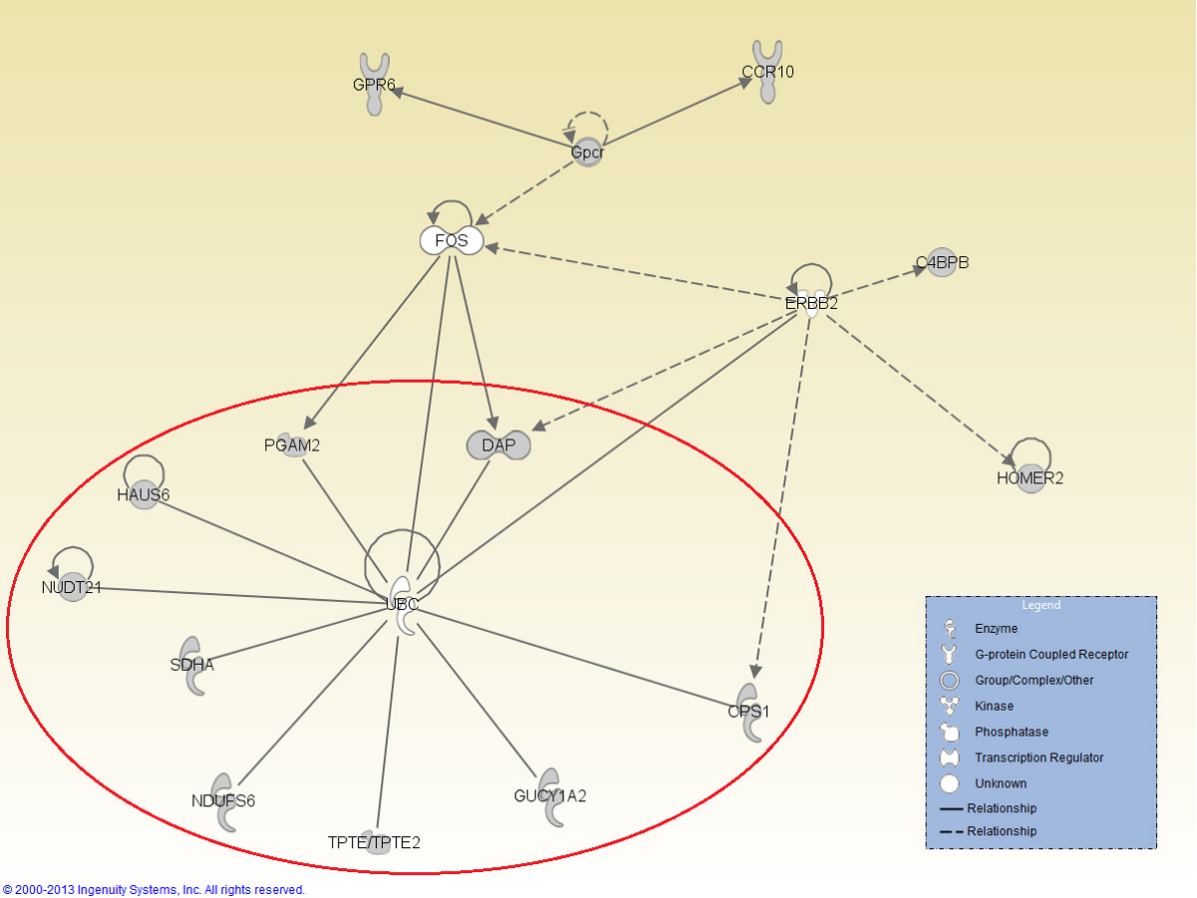
**IPA network visualization of BiNets 52 and 228**. A network found by analyzing the combined network of BiNets 52 and 228 using IPA. The sub network within the red circle is the UBC network whose survival test result is shown in Figure 4.

**Figure 4:**
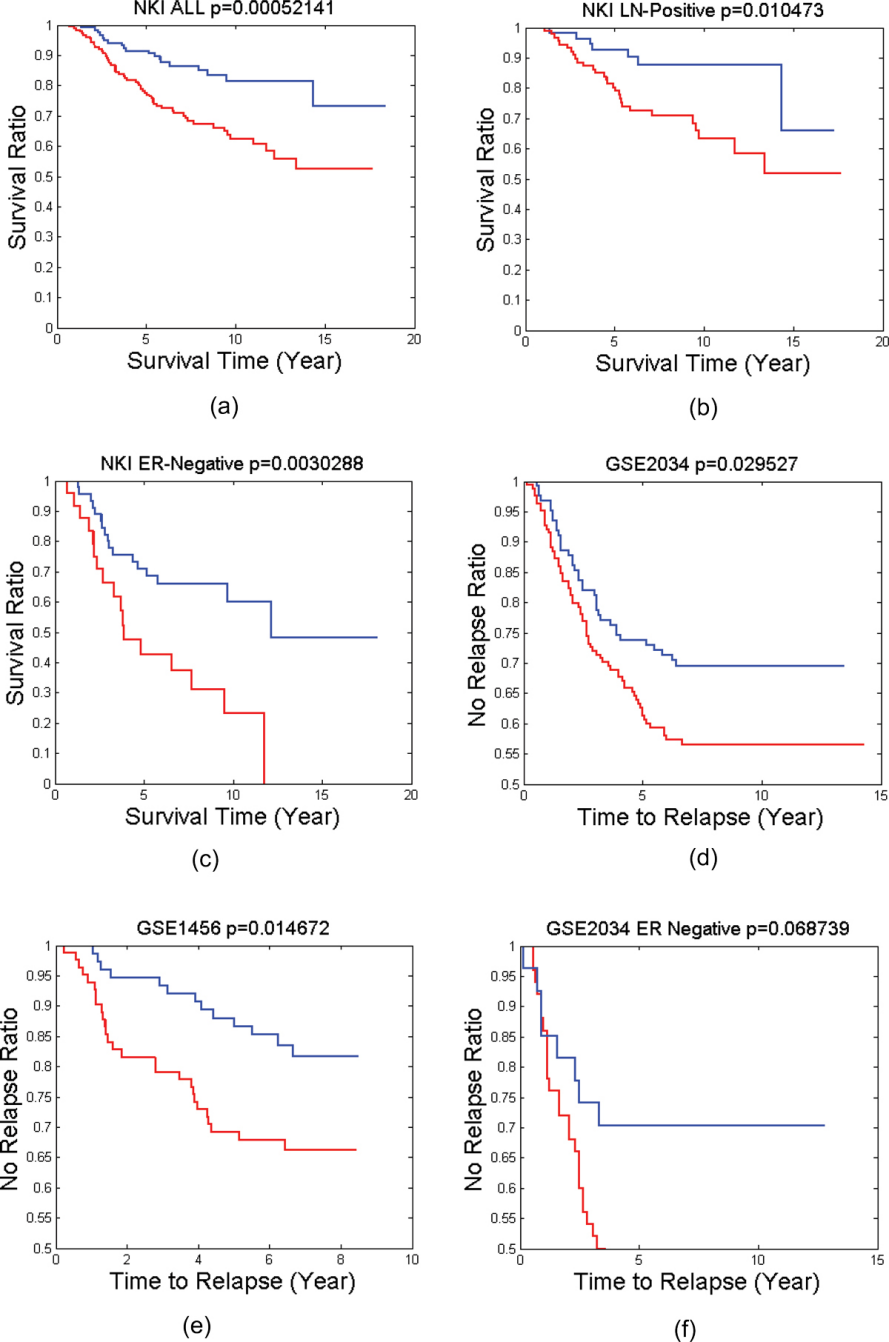
**Survival tests of the UBC network**. The survival results of UBC Network (containing genes "UBC, DAP, CPS1, GUCY1A2, TPTE/TPTE2, NDUFS6, SDHA, NUDT21, HAUS6, PGAM2") on (a) All patients in NKI dataset (b) LN-Positive patients in NKI dataset, (c) ER-Negative patients in NKI dataset, (d) All patients in GSE2034 datasets, (e) All patients in GSE1456 dataset, (f) ER Negative patients in GSE2034 dataset. Blue lines are the survival curves of good survival groups. Red lines are the survival curves of poor survival groups.

For each patient group or subtype, we have identified a number of BiNets with p-value less than 0.05 in log rank test as shown in Table 1. Many of them are good candidates for breast cancer prognosis in the corresponding patient group or subtype. In the past we have also successfully identified potential biomarkers for such patient groups [16,25]. However, identifying good biomarkers for prognosis on ER-negative breast cancer remains a challenge. In this work, we successfully identified several BiNets with strong ER-negative prognosis power. Among them, two BiNets (BiNet 52 and BiNet 228) have both low p-values and well-separated survival curves (Figure 2). We searched for interactions for genes from the two BiNets in IPA Knowledge Base, and found both BiNets contain genes surrounding the gene *UBC*, although *UBC *is not included in either BiNet. We wanted to find out if a combination of the two BiNets will reinforce their prognostic power in the survival test. Thus, we conducted another survival test on the combined gene list of the two BiNets plus *UBC*. It resulted in an even better separation of the ER-negative patient outcomes with a p-value of 4.924*E *− 5, as shown in Figure 2(d), whereas the breast cancer prognosis benchmark van't Veer-70 genes virtually has no prognosis power at all. By further examining the interactions among the genes in this BiNet using IPA (Figure 3), we obtain a gene interaction network centered on the *UBC *gene that possesses a strong prognosis power in survival tests on nearly all types of patient groups tested in this work (Figure 4).

## Discussion and conclusion

As shown in Table 2 the ten macro bipartite networks cover many key biological processes including cell cycle, immune response, cell-cell signaling, respiratory electron transport chain, and defense response to virus. Despite the size difference between the tumor side and the stroma side in these macro bipartite networks, top enriched Biological Process (BP) terms are often shared between the two sides. This indicates that the underlying biological processes are synchronized between tumor and stroma, presumably via cell-cell signaling mechanisms.

An interesting exception is the 3-rd macro bipartite network. The genes in the stroma side are enriched with the biological process of "development of primary male sexual characteristics" and "male sex differentiation". These male-gender specific GO terms seem to be inconsistent with the fact that the data were obtained from female breast cancer patients. A detailed inspection on the genes in this macro bipartite network indicates that it actually contains several key sex hormone related genes such as ESR1 (estrogen receptor *α*) and AR (androgen receptor) as well as ERBB4. These genes are all well known for their involvement with breast cancer prognosis [26-28]. The fact that the gene expression ESR1 shows high correlation between tumor and stroma suggests that estrogen, an important factor in breast cancer development, not only affects the tumor epithelial cells but may also affect the stroma cells in similar ways. Since ESR1 is a target for breast cancer drugs such as tamoxifen, it is thus important to study the effect of the drugs on the stroma cells such as fibroblast in addition to the cancer cells. Therefore a more comprehensive characterization of the drug effects and mechanisms can be pictured.

As shown in Table 1, there are a good number of BiNets that can separate patient cohorts from different breast cancer microarray studies into two subgroups with significant differences. Among them, some can achieve highly significant prognosis with very small p-values. In the past studies we have also successfully identified gene lists that demonstrate good prognostic power in survival tests [16,25]. But such discoveries on ER-negative patients are quite limited. Thus in this work, we are particularly interested in finding BiNets that are potential biomarkers for ER-negative patients.

The strong prognostic power of the combined BiNets on ER-Negative patients led us to hypothesize that *UBC *and its interacting genes play an important role in breast cancer prognosis. To test our hypothesis, we extracted a *UBC *network, which consists of the *UBC *gene and directly interacting genes (i.e., genes that have PPI with the *UBC *gene in BiNets 52 and 228), as shown in the red circle of Figure 3. Then we applied this gene network to survival analysis on all 5 patient cohorts (NKI ALL, NKI LN-Positive, NKI ER-Negative, GSE2034, GSE1456), and it generated p-values less than 0.05 in all of them (Figure 4(a-e)). We also tested it on the ER-negative group of GSE2034, and we also get a p-value quite close to 0.05 (Figure 4(f)). To the best of our knowledge, this is the first report of discovering a gene list that has significant prognosis results on all major subtypes of breast cancer and their mixture.

Our observation is further supported by the recent research on ubiquitin and cancers. Ubiquitin is a small regulatory protein that can be attached to proteins and label them for destruction. *UBC *is the gene encodes Ubiquitin C protein. It is known that many proteins studied by clinical breast cancer researchers, such as cyclins, CDK inhibitors, and the SCF in cell cycle control, are involved in ubiquitin pathways [29]. In addition, Mani and Gelmman [30] discovered that ubiquitin plays a critical role in protein degradation pathways, which are targets for cancer therapy. Our discovery provides biologists and clinicians an additional promising hypothesis that the *UBC *gene network is effective in the prognosis of multi types of breast cancers. Based on the previous discoveries [29,30], we conjecture that the *UBC *gene network is also a promising target for cancer therapy.

In summary, we developed a bipartite subnetwork component mining algorithm BCM for weighted bipartite graphs and applied it to mine the interaction networks between the breast cancer tumor and its microenvironment. Our results reveal highly coordinated biological processes such as cell cycle and immune responses between tumor and stroma. In addition, we identified potential biomakers which can perform very well on the ER-negative type of breast cancer prognosis.

## Materials and methods

### Datasets

GSE5847 was used to construct the tumor-stroma coexpression network. NKI dataset [20,21], GSE1456 (Stockholm) dataset [22], and GSE2034 (Wang) dataset [23,24] were used to perform survival tests.

### Microarray data processing

Gene expression microarray dataset GSE5847 was obtained from the NCBI Gene Expression Omnibus. It contains 95 gene expression profiles on Affymetrix HU133 Plus 2.0 genechip from 48 patients. Out of them, 47 pairs of matched tumor and stroma samples were used in our study. Since the tumor and the stroma datasets are normalized separately, an additional linear global normalization between microarray data for the two tissues were performed. This normalization does not change the rank or linear relationship between any pair of genes except to match the median gene expression levels from all the probes.

Genes with small (*<*20%) variation in expression profiles were excluded, since low variation will lead to bias in correlation coefficient computing. Only probes with available matched gene names were used.

### Construction of tissue-tissue gene co-expression network

**Figure 5:**
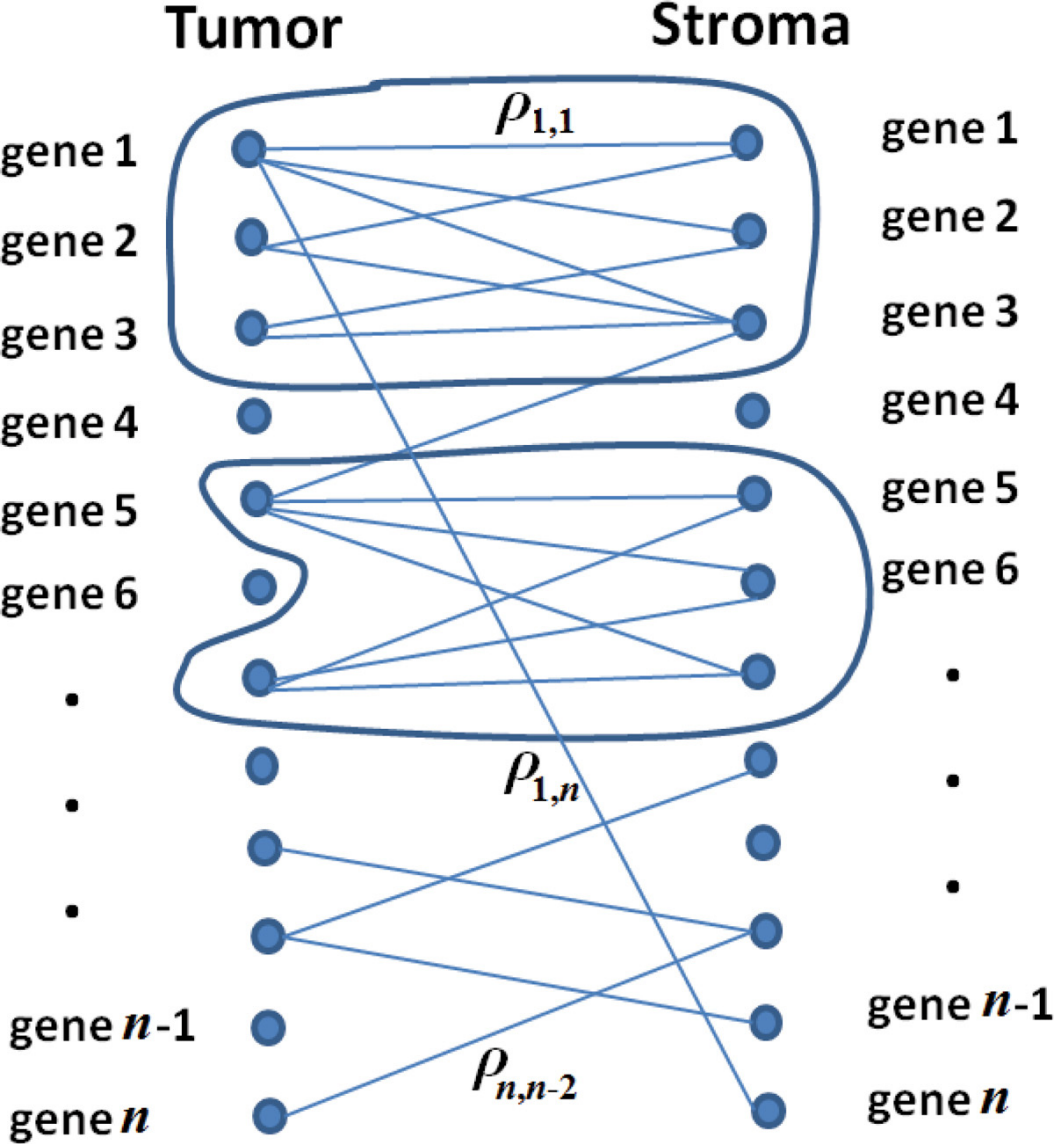
**Bipartite graph of the tissue-tissue network**. An illustration of the formulation of the tissue-tissue network as a weighted bipartite graph. For clarity of the figure, we do not show all the edges. Bipartite sub-networks in the two circles are examples of BiNets.

Given a set of *K *samples with two types tissues, we compute the Pearson correlation coefficient (*ρ_i,j_*) between any gene *g_i _*in tissue 1 and gene *g_j _*in tissue 2. As shown in Figure 5, a bipartite graph between the two tissues can thus be established. In this graph, nodes are the genes in both tissues. The weight of an edge is defined as the Pearson correlation coefficient (*ρ*) between the expression profiles for the two genes connected by the edge (for easy visualization, we did not show all the edges in Figure 5). Our goal is to identify densely-connected-bipartite components (i.e., bipartite sub networks) of the weighted bipartite graph.

### Bipartite sub network mining with bounded density gurantee

Let $G=\left(V_{X},\;V_{Y},\;E\right)$ denote a bipartite graph with set $V_{X}$ of vertices on one side, and set $V_{Y}$ of vertices on the other side. *E *is the set of edges connecting vertices between $V_{X}$ and $V_{Y}$. $B=\left(V_{X},V_{Y},E\left(B\right)\right)$ is a BiNet of a bipartite graph $G=\left(V_{X},\;V_{Y},\;E\right)$ if and only if $V_{X}\subseteq V_{X}$ and $V_{Y}\subseteq V_{Y}$, and *E*(*B*) be the set of edges induced by *V_X _*and *V_Y _*on *G*.

Let *w*(*e*) be the weight of an edge *e *∈ *E*. Let *a *= |*V_X_*|, *b *= |*V_Y_*|. We define the density of *B *to be: $d\left(B\right)=\frac{\sum_{e\in E\left(B\right)}w\left(e\right)}{ab}$. It is easy to see *d*(*B*) is the average weight of edges of *B*.

For a vertex $v\in V_{X}-V_{X}$, we define its density contribution to *B *by: $d{\left(v,B\right)}_{X}=\frac{\sum_{u\in \;V_{Y}}w\left(uv\right)}{b}$. Similarly, for a vertex $v\in V_{Y}-V_{Y}$, we define its density contribution to *B *by: $d{\left(v,B\right)}_{Y}=\frac{\sum_{u\in \;\;V_{X}}w\left(uv\right)}{a}$. A key idea of our algorithm is to grow a dense bipartite component by iteratively adding high contribution vertices from either side that can result in a density bound guarantee.

The pseudocode of our bipartite subnetwork component mining algorithm (BCM) is given in Algorithm 1. BCM discovers a BiNet by starting from an unselected edge with weight no less than a threshold. The density of the discovered BiNet is guaranteed to be bounded by a constant factor of the weight of the starting edge. The purpose of starting from an unselected edge is to avoid excessive numbers of highly overlapped BiNet. However, it shall be noted that unlike traditional clustering methods (such as *k*-means), BCM allows a vertex to be shared by multiple BiNets. It is also necessary to point out that quasi-clique (which resembles a fully connected graph) mining algorithm with bounded density known as QCM is available for weight graphs [31], which hints us to develop BCM. However, the QCM algorithm and its properties are not readily extendable to weighted bipartite graphs.

**Algorithm 1 **$BCM\left(G\;=\left(V_{X},V_{Y},E\right),\;\alpha_{a},\;\alpha_{b},\;\beta \right)$

1: Sort *E *such that edges in *E *are ranked in descending order of their weights;

2: Let *w_max _*be the weight of the first edge in *E*.

3: *Selected *= ∅

4: **for all ***e *= (*x*, *y*) ∈ *E ***do**

5:     **if ***w*(*e*) *< βw_max _***then**

6:         break;

7:     **end if**

8:     **if ***e *∈ *Selected ***then**

9:         continue;

10:     **end if**

11:     B=∅;

12:     Create an empty biclique *B *= (*V_X_*, *V_Y_*);

13:     *V_X _*= *V_X _*∪ {*x*}; *V_Y _*= *V_Y _*∪ {*y*};

14:     **while **true **do**

15:         Pick $p\in V_{X}-V_{X}$ such that $d{\left(p,B\right)}_{X}$ is maximum;

16:         Pick $q\in V_{Y}-V_{Y}$such that $d{\left(p,B\right)}_{Y}$ is maximum;

17:         **if **$d{\left(p,B\right)}_{X}>d{\left(q,B\right)}_{Y}$**then**

18:             **if **$d{\left(p,B\right)}_{X}\geq \alpha_{a}d\left(B\right)$**then**

19:                 *V_X _*= *V_X _*∪ {*p*};

20:                 Insert into *Selected *any edge in *E *connecting *p *to any vertex in *V_Y_*;

21:             **else**

22:                 break;

23:             **end if**

24:         **else**

25:             **if ***d*(*q*, *B*)*_Y _*≥ *α_b_d*(*B*) **then**

26:                 *V_Y _*= *V_Y _*∪ {*q*};

27:                 Insert into *Selected *any edge in *E *connecting *q *to any vertex in *V_X_*;

28:             **else**

29:                 break;

30:             **end if**

31:         **end if**

32:     **end while**

33:     B=B∪{B};

34: **end for**

36: **return **;

In BCM, we set $\alpha_{a}=1-\frac{1}{\lambda_{a}\left(a+\tau +1\right)}$, and $\alpha_{b}=1-\frac{1}{\lambda_{b}\left(b+\tau +1\right)}$, where $\lambda_{i}=\text{max}\left\{1,\frac{C}{{\left(i+\tau +1\right)}^{2}}\right\}$. We use two parts in the following to show that every bipartite subgraph B∈B, which is outputted by Algorithm 1, has a bounded density.

In the first part, we analyze the density ratio between two consecutive steps of BCM. Let $f\left(a,b\right)=d\left({B_{a}}_{b}\right)=\frac{\sum_{e\in \;E\left(B{\;}_{ab}\right)}w\left(e\right)}{ab}$ be the density of *B_ab_*, a transit bipartite subgraph in Algorithm 1, with *a *number of vertices in *V_X _*and *b *number of vertices in *V_Y_*. Adding one more vertex *v *to *B_ab _*by Algorithm 1 will make the density of *B_ab _*be either *f*(*a *+ 1, *b*) or *f*(*a*, *b *+ 1). Without loss of generality, let us assume the new vertex *v *is added to *V_X _*and the density of *B_ab _*becomes *f*(*a *+ 1, *b*). According to Algorithm 1, we have

$$d{\left(v,B\right)}_{X}\geq \alpha_{a}d\left(B\right)$$

which is equivalent to:

(1) $$\frac{\sum_{u\in \;V_{Y}}w\left(uv\right)}{b}\geq \alpha_{a}f\left(a,b\right)$$

(1) can be rewritten as

(2) $$\frac{\left(a+1\right)bf\left(a+1\;,b\right)-abf\left(a,b\right)}{b}\geq \alpha_{a}f\left(a,b\right)$$

From (2) we have

(3) $$\left(a+1\right)f\left(a+1,\;b\right)\geq af\left(a,\;b\right)+\alpha_{a}f\left(a,\;b\right)$$

Thus, we have

(4) $$\frac{f\left(a+1,b\right)}{f\left(a,b\right)}\geq \frac{a+\alpha_{a}}{a+1}=\frac{\lambda_{a}\left(a+1\right)\left(a+\tau +1\right)-1}{\lambda_{a}\left(a+1\right)\left(a+\tau +1\right)}$$

With similar analysis, we also have

(5) $$\frac{f\left(a,b+1\right)}{f\left(a,b\right)}\geq \frac{b+\alpha_{b}}{b+1}=\frac{\lambda_{b}\left(b+1\right)\left(b+\tau +1\right)-1}{\lambda_{b}\left(b+1\right)\left(b+\tau +1\right)}$$

Next we show that a bipartite subgraph B∈B has a bounded density with respect to the weight of the starting edge. Assume *B *= (*V_X_*, *V_Y_*, *E*(*B*)) where |*V_X_*| = *s *and |*V_Y_*| = *t*. Thus, the density of *B *is *f*(*s*, *t*). According to Algorithm 1, the density of *B *evolves from *f*(1, 1) to *f*(*s*, *t*), with a vertex added to either *V_X _*or *V_Y _*in each step. To show that the density of *B *is bounded, we only need to show that $F=\frac{f\left(s,t\right)}{f\left(1,1\right)}$ is a constant.

**Theorem 1**. *Let f*(*s*, *t*) *be the density of BiNet B *= (*V_X_*, *V_Y_*, *E*(*B*)) *where *|*V_X_*| = *s and *|*V_Y_*| = *t. Let f *(1, 1) *be the weight of the starting edge for B in Algorithm *BCM*. Then F *= *f*(*s*, *t*)*/f*(1, 1) *is larger than *${\left(\frac{2C-\tau -2}{2C}\right)}^{2\left\lceil \sqrt{C}-\tau -2\right\rceil }\times {\left(\frac{\left\lceil \sqrt{C}-\tau -1\right\rceil }{\left\lceil \sqrt{C}-\tau \right\rceil }\right)}^{2}$, *where C and τ are nonnegative integer parameters*.

(See Appendix for proof.)

*C *and *τ *are used for tuning the bound. For example, if we choose *C *= 100 and *τ *= 1, according to Theorem 1, we have:

$$F>\;{\left(\frac{200-1-2}{200}\right)}^{2*7}\times {\left(\frac{10-1-1}{10-1}\right)}^{2}\approx 0.64$$

One can easy to get a large bound by setting a large *C *and a small *τ*. For example, when *C *= 10000 and *τ *= 0, we have *F >*0.96 by (14).

### Evaluation of gene networks as potential prognostic biomarkers

Once the bipartite graph between the tumor genes and stromal genes was constructed, it was subjected to the BCM algorithm for BiNet discovery. We set the parameters *C *= 36, *τ *= 2 which guarantees *F >*47.4% according to Theorem 1. We also set *β *= 0.7 to ensure a reasonably large search space. After this step, we map the BiNets back to genes. Each BiNet is also corresponding to *one combined gene set *which is obtained by union the two separate gene sets in the BiNet into one.

For genes in each BiNet, their potential as breast cancer prognostic biomarker was tested using breast cancer microarray datasets. The primary dataset used for testing is the well known NKI dataset which are composed of 295 patients. To validate the survival analysis results, two more microarray datasets from GEO were used: GSE1456 containing data for 159 breast cancer patients, and GSE2034 dataset containing data for 286 breast cancer patients. The time-to-recurrence information for patients from these two datasets were used in the survival analysis.

In the survival test, genes in each BiNet (including both tumor and stroma sides) are used as features for the patients. The patients are then divided into two groups based on these feature values by K-means algorithm (K = 2, distance=cityblock, repeating 100 times). Log-rank test (publicly available at: http://www.mathworks.nl/matlabcentral/fileexchange/20388) was used to determine the statistical significance (p-value) between the survival time (or time-to-recurrence) for the two group of patients.

### Summarize BiNets into macro bipartite networks by merging

In order to understand the general structure of the tumor-stroma network, BiNets were further summarized into a few macro bipartite networks by SINGEMERGE[25], a network merge algorithm that guarantees merge density. We set the density threshold to be 0.3 and we merged the 372 BiNets into 10 macro bipartite networks which were further subjected to gene ontology enrichment analysis. Figure 1 is the merging dendrogram and the parameters of the 10 macro bipartite networks including their GO analysis are listed in Table 2.

### Gene Ontology (GO) analysis

GO enrichment analysis for the gene list from each macro bipartite network is carried out using ToppGene (http://toppgene.cchmc.org/enrichment.jsp), a publicly available web tool. Pathway analysis on selected BiNets is further carried out using Ingenuity Pathway Analysis (https://analysis.ingenuity.com).

## Appendix

Proof of Theorem 1

*Proof*. To facilitate our discuss, we assume it takes *n *steps to generate *B *where *f_k _*denotes the density of *B *at step *k*, e.g., *f*_1 _= *f*(1, 1) and *f_n _*= *f*(*s*, *t*). Thus

(6) $$F=\frac{f_{n}}{f_{1}}=\frac{f_{2}}{f_{1}}\frac{f_{3}}{f_{2}}\cdots \frac{f_{n-1}}{f_{n-2}}\frac{f_{n}}{f_{n-1}}$$

For some $\frac{f_{k+1}}{f_{k}}$, the change is on *V_X _*thus we apply (4); for others, the change is on *V_Y _*and we apply (5). Let *g_i _*denote the fraction of the new density over the old one when it is the *i*th time of adding a vertex to *V_X_*. Similarly, let *h_j _*denote the fraction of the new density over the old one when it is the *j*th time of adding a vertex to *V_Y_*. Thus (6) can be rewritten as:

(7) $$F=\frac{f_{n}}{f_{1}}=\overset{s}{\underset{i=1}{\prod }}g_{i}\overset{t}{\underset{j=1}{\prod }}h_{t}$$

To analyze (7), we first consider $\prod_{i=1}^{s}g_{i}$, which can be factorized into two parts:

(8) $$\overset{s}{\underset{i=1}{\prod }}g_{i}=\overset{\left\lceil \sqrt{C}-\tau -2\right\rceil }{\underset{i=1}{\prod }}g_{i}\times \overset{s}{\underset{i=\left\lceil \sqrt{C}-\tau -1\right\rceil }{\prod }}g_{i}$$

Given $\lambda_{i}=\text{max}\left\{1,\;\frac{C}{{\left(i+\tau +1\right)}^{2}}\right\}$ and (4), we have:

(9) $$\begin{array}{c} \overset{\left\lceil \sqrt{C}-\tau -2\right\rceil }{\underset{i=1}{\prod }}g_{i}=\overset{\left\lceil \sqrt{C}-\tau -2\right\rceil }{\underset{i=1}{\prod }}\frac{C\left(\frac{i+1}{i+\tau +1}\right)-1}{C\left(\frac{i+1}{i+\tau +1}\right)}\\ \;\;\;\;\;\;\geq {\left(\frac{2C-\tau -2}{2C}\right)}^{\left\lceil \sqrt{C}-\tau -2\right\rceil } \end{array}$$

(10) $$\begin{array}{c} \overset{}{\prod_{i=\left\lceil \sqrt{C}-\tau -1\right\rceil }^{s}g_{i}}\;=\prod_{i=\left\lceil \sqrt{C}-\tau -1\right\rceil }^{s}\frac{(i+1)(i+\tau +1)-1}{\left(i+1)(i+\tau +1\right)}\;\\ \;\;\;\;\;\;\;\;\;\;\;\;\;\;\;\;\;\;\;\;\;\;\;\;\;\;\;\;\;\;\;\;\;\;\;\;\geq \prod_{i=\left\lceil \sqrt{C}-\tau -1\right\rceil }^{s}\frac{{\left(i+1\right)}^{2}-1}{{\left(i+1\right)}^{2}} \end{array}$$

Let *Mi *= (*i *+ 1)^2 ^− 1 and *N_i _*= (*i *+ 1)^2^, then we have $\frac{M_{i}}{N_{i-1}}=\frac{{\left(i+1\right)}^{2}-1}{i^{2}}=\frac{i+2}{i}$. Thus, (10) can be further extended as:

(11) $$\begin{array}{c} \overset{s}{\underset{i=\left\lceil \sqrt{C}-\tau -1\right\rceil }{\prod }}g_{i}\\ \;\;\;\;\;\;\;\;\;\;\;\;\;\;\;\;\geq \overset{s}{\underset{i=\left\lceil \sqrt{C}-\tau -1\right\rceil }{\prod }}\frac{{\left(i+1\right)}^{2}-1}{{\left(i+1\right)}^{2}}\\ \;\;\;\;=\overset{s}{\underset{i=\left\lceil \sqrt{C}-\tau -1\right\rceil }{\prod }}\frac{M_{i}}{N_{i}}\\ \;\;\;\;\;\;\;\;\;\;\;\;\;\;\;\;\;\;\;\;\;\;\;\;\;\;\;\;\;\;\;\;\;\;\;\;\;\;\;\;\;\;\;=\frac{{\left(\left\lceil \sqrt{C}-\tau -1\right\rceil +1\right)}^{2}-1}{\left(\left\lceil \sqrt{C}-\tau -1\right\rceil +1\right)\left(\left\lceil \sqrt{C}-\tau -1\right\rceil +2\right)}\frac{\left(s+1\right)\left(s+2\right)}{{\left(s+1\right)}^{2}}\\ >\frac{\left\lceil \sqrt{C}-\tau -1\right\rceil }{\left\lceil \sqrt{C}-\tau \right\rceil } \end{array}$$

Combining (8), (9), and (11), we have

(12) $$\overset{s}{\underset{i=1}{\prod }}g_{i}>{\left(\frac{2C-\tau -2}{2C}\right)}^{\left\lceil \sqrt{C}-\tau -2\right\rceil }\frac{\left\lceil \sqrt{C}-\tau -1\right\rceil }{\left\lceil \sqrt{C}-\tau \right\rceil }$$

Using the similar analysis as above, we have:

(13) $$\overset{t}{\underset{j=1}{\prod }}h_{j}>{\left(\frac{2C-\tau -2}{2C}\right)}^{\left\lceil \sqrt{C}-\tau -2\right\rceil }\frac{\left\lceil \sqrt{C}-\tau -1\right\rceil }{\left\lceil \sqrt{C}-\tau \right\rceil }$$

Combining (12) and (13), we eventually have the bound for *F*:

(14) $$\begin{array}{c} \;\;\;\;\;\;\;\;\;\;\;\;\;\;\;\;\;\;\;\;\;\;\;\;\;\;\;\;\;\;\;\;F=\frac{f_{n}}{f_{1}}=\overset{s}{\underset{i=1}{\prod }}g_{i}\overset{t}{\underset{j=1}{\prod }}h_{t}\\ >{\left(\frac{2C-\tau -2}{2C}\right)}^{2\left\lceil \sqrt{C}-\tau -2\right\rceil }\times {\left(\frac{\left\lceil \sqrt{C}-\tau -1\right\rceil }{\left\lceil \sqrt{C}-\tau \right\rceil }\right)}^{2} \end{array}$$

(To facilitate our analysis, *C *is set to be larger than (*τ *+ 2)^2^.)

## Competing interest

The authors declare that they have no competing interests.

## Authors' contributions

YX led the algorithm design and network analysis. JZ carried out the initial study and led the result analysis from the view of cancer biology. KH led the project including development of the idea, data selection and preprocessing, and the development of the whole workflow. All authors edited the manuscript.

## Declarations

This work was supported in part by the National Science Foundation under Grant #1019343 to the Computing Research Association for the CIFellows Project, and by the National Institutes of Health under Grant NCI R01CA141090.

This article has been published as part of *BMC Genomics *Volume 14 Supplement 5, 2013: Twelfth International Conference on Bioinformatics (InCoB2013): Computational biology. The full contents of the supplement are available online at http://www.biomedcentral.com/bmcgenomics/supplements/14/S5.
